# *RPE65* c.393T>A, p.(Asn131Lys): Novel Sequence Variant Detected

**DOI:** 10.1155/2022/5710080

**Published:** 2022-04-01

**Authors:** Mirjana Bjeloš, Mladen Bušić, Ana Ćurić, Damir Bosnar, Borna Šarić, Leon Marković, Biljana Kuzmanović Elabjer, Benedict Rak

**Affiliations:** ^1^Department of Ophthalmology, Reference Center of the Ministry of Health of the Republic of Croatia for Pediatric Ophthalmology and Strabismus, University Hospital “Sveti Duh”, Zagreb, Croatia; ^2^Faculty of Medicine, Josip Juraj Strossmayer University of Osijek, Osijek, Croatia; ^3^Faculty of Dental Medicine and Health Osijek, Josip Juraj Strossmayer University of Osijek, Osijek, Croatia

## Abstract

**Background:**

Leber congenital amaurosis (LCA) is a monogenic, but genetically heterogenous disease, and at least 27 genes are implicated. This case report is aimed at providing evidence to link the novel variant *RPE65* c.393T>A, p.(Asn131Lys), variant of uncertain significance (VUS), to clinical phenotype and to set the ground for objective assignment of pathogenicity confidence. *Case Presentation*. A case report of a female patient with LCA who manifested with nystagmus, night blindness, profound visual deficiency, and peripheral involvement of the retina consistent with *RPE65* dystrophy. A thorough clinical examination, diagnostic evaluation, and genetic testing were performed. The patient was a compound heterozygote in *trans* form: *RPE65* c.304G>T, p.(Glu102∗) pathogenic, and *RPE65* c.393T>A, p.(Asn131Lys), VUS. The latter variant is absent in healthy controls and is considered harmful on in silico prediction.

**Conclusions:**

We conclude that *RPE65* c.393T>A, p.(Asn131Lys) contributed to the pathologic phenotype, demonstrating its significance clearly in the case presented, and should be reclassified according to the criteria of evidence as likely pathogenic. This being the case, patients with this specific variant are likely candidates for genetic treatment.

## 1. Introduction

Leber congenital amaurosis (LCA) is one of the most common causes of blindness in children with incidence of 2 to 3 per 100 000 newborns [1], characterized by early and severe visual loss occurring usually before the age of 1 year, nystagmus, sluggish or almost absent pupillary reflexes, and reduced or flattened electroretinogram (ERG) [2].

LCA is a monogenic, but genetically heterogeneous disease, and around 27 genes are implicated [3] and inherited in an autosomal recessive manner, except for *IMPDH1* linked to dominant transmission and *CRX* known to cause either dominant or recessive disease [2, 3]. Among the known disease-causing genes, *RPE65* mutations were first identified with the prevalence ranging from 1.7% to 16% in LCA cohorts in the United States, Canada, Saudi Arabia, Asia, and India, with most cases occurring in Western populations [4].

RPE65 protein (GenBank accession No. NP000320.1) is an isomerase preferentially expressed in the retinal pigment epithelium (RPE) [4]. It is of utmost importance to define the molecular diagnosis of the patients with LCA due to existing targeted therapeutic option: voretigene neparvovec (Luxturna®, Novartis, Basel, Switzerland). The prevalence of *RPE65* variants in LCA patients has been estimated to 6-8% [5, 6].

This is the first report of the novel variant c.393T>A, p.(Asn131Lys), variant of uncertain significance (VUS), RP, LCA, autosomal recessive. This case report is aimed at providing evidence to link the variant to clinical phenotype and to provide the ground for objective assignment of pathogenicity confidence.

## 2. Case Presentation

A 66-year-old female with inherited retinal dystrophy (IRD) was referred to our clinic in June 2021 for clinical examination and genetic testing. Since infancy, her parents noticed that her eyes are “flickering” and she could not see well in the dark or find toys on the floor in the dark room. Her visual performance was sufficient to enable attendance at regular elementary school until the 7th grade (13 years), when her visual acuity further declined, and was thus enrolled in the Center for the Education of the Blind. At the age of 16, a more pronounced decline in visual acuity ensued, progressively worsening over the years. Her mother and father were healthy, as well as 12 siblings. She has 3 healthy children and 2 healthy grandchildren.

At clinical examination, at 1355 lux of room illumination, her visual acuity (VA) measured 1.0 logMAR binocularly at distance (tested on 3 m) and 1.1 logMAR at near (tested on 40 cm). Right eye (RE) measured 1.5 logMAR on 1 m and 30 cm, while left eye (LE) measured 1.0 logMAR distance (tested on 3 m) and 1.1 logMAR near (tested on 40 cm). She could not perform CSV-1000 contrast sensitivity testing or standardized color vision tests. Tested with A4 colored papers, the patient was able to discriminate red, orange, green, and blue colors clearly, while purple color could not be distinguished from blue. Octopus® (Haag-Streit Inc, Mason (OH), USA) static G1 perimetry evidenced markedly reduced retinal sensitivity within the central 30°: mean deviation measured 26.5 dB on the RE and 26.2 dB on the LE, while mean sensitivity measured 0.0 dB and 0.3 dB, respectively. MAIA microperimetry (iCare Finland Oy, Vantaa, Finland) was attempted, but the patient failed to fixate the test badge with either eye due to low VA and nystagmus. Central foveal thickness analysed by optical coherence tomography (HRA+ OCT Spectralis®, Heidelberg Engineering, Heidelberg, Germany) measured 258 and 251 microns in the RE and LE, respectively.

**Figure 1:**
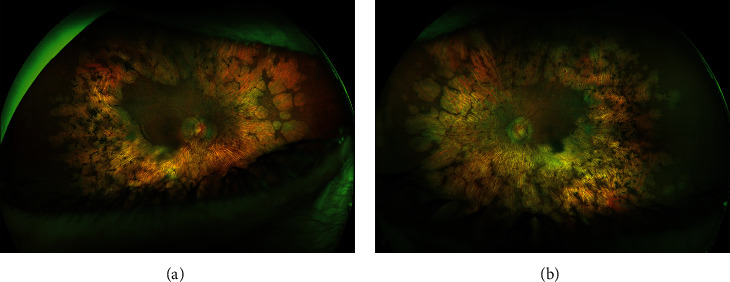
ultra-widefield fundus photos of the (a) right eye and the (b) left eye showing pale and waxy optic nerve head with clear boundaries and area of atrophy peripapillary, paving-stone degeneration, drusen-like retinal deposits, bone spicule-like pigmentary clumping, and attenuated retinal vessels at mid- and far periphery.

**Figure 2:**
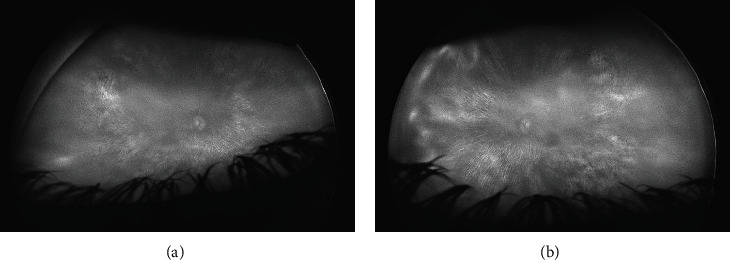
Fundus autofluorescence photos of the (a) right eye and the (b) left eye. Pale fundi and absence of RPE autofluorescence. Chorioretinal atrophy reveals the hyperautofluorescent attributes of the sclera.

Optos® California (Optos Inc, Marlborough, MA, USA) ultra-widefield imaging depicted pale and waxy optic nerve head with clear boundaries and area of atrophy peripapillary. The macula was mostly atrophic; however, wider areas without atrophy larger than 3-disc diameter were present bilaterally. Diffuse patches of paving-stone degeneration, drusen-like retinal deposits, bone spicule-like pigmentary clumping, and attenuated retinal vessels were observed at the mid- and far periphery (Figure 1). Fundus autofluorescence was absent (Figure 2).

Electrophysiological testing (Roland Consult RETI-port/scan 21, Roland Consult Stasche & Finger GmbH–German Engineering, Brandenburg an der Havel, Germany) according to ISCEV standards was further performed. Full-field ERG bilaterally depicted barely detectable scotopic and photopic responses. Retinoscopy in cycloplegia did not detect significant refractive error: RE: +1.50/−0.50 × 50°, and LE: +1.50/−0.50 × 120°.

The patient's buccal swab was collected for genetic testing for mutations in the *RPE65* gene and sent to Blueprint Genetics Laboratory, Espoo, Finland.

The Blueprint Genetics Retinal Dystrophy Panel (version 6, 2020, Feb 22) Plus sequence analysis and copy number variation analysis identified a heterozygous nonsense variant *RPE65* c.304G>T, p.(Glu102∗) and a heterozygous missense variant *RPE65* c.393T>A, p.(Asn131Lys) [7]. The Next-Generation Sequencing data indicated that these variants were on different parental alleles (in *trans*) in this patient.

## 3. Discussion

### 3.1. *RPE65* c.304G>T, p.(Glu102∗)

This pathological variant is linked to LCA type 2 phenotype [8]. Clinical features of the disease include the following: blindness, cataract, decreased light- and dark-adapted ERG amplitude, fundus atrophy, keratoconus, photophobia, RP, and reduced VA [3, 9]. In the Genome Aggregation Database [10], a large reference population database (*n* > 120,000 exomes and >15,000 genomes) which is aimed at excluding individuals with severe pediatric disease, 9 individuals heterozygous for the variant and no homozygotes were found in the dataset [10]. The variant is a cause of a premature stop codon in exon 4 (of 14 total exons) and leads to loss of normal protein function, either through protein truncation or nonsense-mediated mRNA decay [8]. The *RPE65* c.304G>T, p.(Glu102∗) has been reported in a homozygous state in two siblings with LCA [11] and together with c.271C>T, p.(Arg91Trp) in two patients with autosomal-recessive *RPE65-*related IRD [12].

### 3.2. *RPE65* c.393T>A, p.(Asn131Lys)

This missense variant predicts asparagine (uncharged polar, hydrophilic) and lysine (basic) substitution resulting in changes in charge. This variant is absent in gnomAD [10]. The mutation targets high evolutionary conserved nucleotide in the carotenoid oxygenase domain of the protein. Grantham score of 94 affirms moderate physicochemical difference between asparagine and lysine. Muttaster, PolyPhen, and Sift in silico tools predict this alteration to be disease causing, possibly damaging, and deleterious. To the best of our knowledge, this variant has not been described in the medical literature or reported in disease-related variation databases such as ClinVar or HGMD [8, 13]. The variant is found in *trans* with another pathogenic variant in a patient with *RPE65* specific phenotype (Figure 2). Maternity and paternity segregation analysis could not have been performed to exclude/confirm de novo event as both unaffected parents were deceased.

There are currently more than 230 known variants in *RPE65* annotated as disease-causing in the HGMD Professional variant database (version 2021.1) [13]. Approximately 60% of the variants are explicitly missense variants, while 40% are truncating variants (i.e., nonsense, frameshift, variants affecting splicing, and gross deletions) [9]. The loss of *RPE65* gene function involves various mechanisms including a lower expression level of RPE65 protein, loss of catalytic activity, or rapid degradation of the protein [14, 15].

Although termed VUS, *RPE65* c.393T>A, p.(Asn131Lys) is (a) absent in population databases and (b) detected *in trans* with a pathogenic variant, representing moderate evidence of pathogenicity [16]. Moreover, the following determinants, (a) multiple computational evidence (Muttaster, PolyPhen, and Sift in-silico tools) supports its deleterious effect, affecting a highly conserved amino acid in the carotenoid oxygenase domain of the protein, (b) the variant is present in the *RPE65* in which missense variants are a common mechanism of disease and benign missense variation occur at a low rate, and (b) patient phenotype is highly specific for a disease with a single genetic etiology (Figure 2), conform with the supporting pathogenic criteria [16]. In general, combining 2 moderate and ≥2 supporting criteria the variant *RPE65* c.393T>A, p.(Asn131Lys) could be reclassified as likely pathogenic [16].

Our patient is a compound heterozygote in *trans* form and manifests disease with all characteristics typical for the *RPE65* gene mutation [17]. Therefore, we conclude that the variant *RPE65* c.393T>A, p.(Asn131Lys) contributed to the pathologic phenotype, demonstrating its significance clearly in the case presented, and should be reclassified according to the criteria of evidence as likely pathogenic.

This being the case, patients with this specific variant in homozygous or compound heterozygous form would be likely candidates for genetic treatment based on recombinant adeno-associated virus vector providing a working *RPE65* gene to act in place of a mutated *RPE65* gene [18].

## Data Availability

The data that support the findings of this study are available from the corresponding author, upon reasonable request.
